# Natural cutaneous anthrax infection, but not vaccination, induces a CD4^+^ T cell response involving diverse cytokines

**DOI:** 10.1186/s13578-015-0011-4

**Published:** 2015-04-26

**Authors:** Rebecca J Ingram, Stephanie Ascough, Catherine J Reynolds, Gökhan Metan, Mehmet Doganay, Les Baillie, Diane E Williamson, John H Robinson, Bernard Maillere, Rosemary J Boyton, Daniel M Altmann

**Affiliations:** Centre for Infection and Immunity, Queen’s University Belfast, Belfast, UK; The Pirbright Institute, Compton, Berkshire UK; Department of Medicine, Imperial College, Hammersmith Hospital, Du Cane Rd, London, W12 ONN UK; Department of Infectious Disease, Erciyes University Hospital, Kayseri, Turkey; The School of Pharmacy and Pharmaceutical Sciences, Cardiff University, Cardiff, UK; Defence Science Technology Laboratory, Porton Down, Salisbury, UK; Institute of Cellular Medicine, Newcastle University, Newcastle upon Tyne, UK; CEA, iBiTecS, Service d’Ingénierie Moléculaire des Protéines (SIMOPRO), Gif Sur Yvette, France

**Keywords:** Anthrax, Cytokine, Lethal factor, T cell, Bacillus anthracis, Vaccination, Infection

## Abstract

**Background:**

Whilst there have been a number of insights into the subsets of CD4^+^ T cells induced by pathogenic *Bacillus anthracis* infections in animal models, how these findings relate to responses generated in naturally infected and vaccinated humans has yet to be fully established. We describe the cytokine profile produced in response to T cell stimulation with a previously defined immunodominant antigen of anthrax, lethal factor (LF), domain IV, in cohorts of individuals with a history of cutaneous anthrax, compared with vaccinees receiving the U.K. licenced Anthrax Vaccine Precipitated (AVP) vaccine.

**Findings:**

We found that immunity following natural cutaneous infection was significantly different from that seen after vaccination. AVP vaccination was found to result in a polarized IFNγ CD4+ T cell response, while the individuals exposed to *B. anthracis* by natural infection mounted a broader cytokine response encompassing IFNγ, IL-5, −9, −10, −13, −17, and −22.

**Conclusions:**

Vaccines seeking to incorporate the robust, long-lasting, CD4 T cell immune responses observed in naturally acquired cutaneous anthrax cases may need to elicit a similarly broad spectrum cellular immune response.

## Findings

### Protective immunity against anthrax

Much research into protective, adaptive immunity against bacterial pathogens has focused largely on the role of neutralising antibodies. There is, however, growing interest in the protective T cell immunity to bacterial infection and the implications of this for rational vaccine design. IL-17A, the hallmark cytokine of the Th17 subset, has been demonstrated to be essential for protection in a number of murine infection models [1]. Recently, it was suggested that although both Th1 and Th17 responses are generated by bacterial infection, the memory Th17 response is short-lived compared to the Th1 response [2]. In the case of *Bacillus anthracis* infection, murine models suggest that protection against anthrax generated by an inactivated spore vaccine is dependent on IFNγ release by Th1 cells [3]. However, the extent to which these effector phenotypes can be extrapolated to natural human infection remains poorly understood.

*B. anthracis* secretes three toxins, Protective Antigen (PA) and two enzymatically active toxin subunits, Lethal Factor (LF) and Edema Factor (EF), which together form tripartite exotoxins, Lethal Toxin (LT) and Edema Toxin (ET) [4]. The two vaccines currently licensed for use in humans, the U.K.-licensed anthrax vaccine precipitated (AVP) and the U.S.-licensed anthrax vaccine adsorbed (AVA or Biothrax), are both derived from a filtered culture supernatant of *B. anthracis* strains [5-7], containing variable amounts of these toxins. Whilst the presence of PA specific toxin neutralizing antibodies is the primary correlate of protection in current human vaccines, reliance upon this antigen alone may limit the promotion of long-lasting memory.

We previously demonstrated long-lived Th1 responses in a cohort of individuals who had either recovered from cutaneous anthrax or were exposed to anthrax toxin components by vaccination [8,9]. Analysis of the cohort of agricultural workers, previously infected with cutaneous anthrax, showed robust CD4^+^ T cell memory to anthrax antigens, in line with the observation that, though occupational exposure is ongoing, reinfection is rarely seen. Despite the fact that the few studies which concern cellular immunity to anthrax have concentrated primarily upon analysing the T cell response to PA [10,11], it is known that both PA and LF are capable of conferring protective immunity in human and animal vaccination studies [4,12]. Protective immunity has been defined by the operational criterion of neutralizing antibody titre, whereas the aim of our work has been to clarify the adaptive immunity correlates of long-term protection at the level of CD4 T cells in survivors of natural exposure.

Our previous work showed that the T cell response to Lethal Factor (LF) was focused upon domain IV [8], this is the catalytic region of the protein and responsible for rapid Mitogen-Activated Protein Kinase (MAPK) cleavage within the host cell. The MAPK pathways are critical in controlling T cell activation and differentiation [13], and through blocking the activation cascade, LT is capable of inhibiting JNK, ERK and p38 mediated T cell proliferation [14,15]. Such inhibition is associated with the reduced production of Th1 cytokines, IFNγ and TNFα, as well as the downregulation of the activation markers, CD69 and CD25 [15,16]. ET is capable of acting in a synergistic manner with LT upon the MAPK pathways to suppress T cell chemotaxis in response to CXCL12 [17], blocking the trafficking of both naïve and effector memory T cells to infected tissues. In combination with the elevation of intracellular cAMP by ET, this has been reported to skew the differentiation of naïve CD4+ T cells towards a Th2 subset, inhibiting activation of Akt1, a protein essential for the development of a Th1 subset, whilst enhancing the activation of the guanine nucleotide exchanger Vav1 and the stress kinase p38 which are involved in Th2 differentiation [18]. Inhibition also impacts on antigen presenting cells (APCs), reducing production of both IFNγ by macrophages, and IL-12 by dendritic cells (DCs) [19,20].

Conversely, recent work has suggested that exposure of human ex vivo cells to ET at low concentrations is capable of promoting a Th17 response [21], and studies in mice have further indicated a key role for IL-17A in protective immunity against inhalational anthrax [22,23]. Human DCs have been found to respond to *B. anthracis* infection by inducing a Th17 response characterised by IL-17 and IFNγ production [24], thus suggesting the involvement of these CD4+ T cells in a protective response. To evaluate the nature of the immune response to *B. anthracis* antigens, and specifically to investigate the possibility of skewing towards certain Th subsets, we assessed cytokine responses of CD4+ T cells against LF domain IV in naturally infected and AVP vaccinated individuals.

## Materials and methods

### Study subjects

Human peripheral blood mononuclear cells (PBMC) were collected from 9 individuals living in an endemic area of Turkey who had a history of cutaneous anthrax within the last 8 years, 10 volunteers from the UK routinely vaccinated every 12 months for a minimum of 4.5 years with the U.K. Anthrax Vaccine Precipitated (AVP) vaccine (U.K. Department of Health) and 10 healthy controls from the UK with no known exposure to anthrax antigens. Previous work has shown that there is no demonstrable difference in healthy controls from the UK and Turkey in terms of HLA or immune cell population responses to anthrax antigens [25,8]. The study was approved by the appropriate ethics committees, (Ericyes University Ethical Committee, UK Department of Health under approval by the Convention on Biological Diversity Independent Ethics Committee for the UK Ministry of Defence, and Ethics REC reference number 08/H0707/173), and was performed in accordance with the 1964 Declaration of Helsinki and its later amendments. All participants gave their informed consent prior to inclusion in the study.

### Antigen stimulation

PBMCs were prepared from the sodium heparinised blood using Accuspin tubes (Sigma-Aldrich) with Histopaque-1077 and centrifuged at 800 *g* for 30 minutes, after which cells were removed from the interface and washed twice in AIM-V serum free media. Cells were counted for viability and resuspended at 2x10^6^ cells/ml, then stimulated for 72 h with 25 μg/ml of LF domain IV (which represented the lowest concentration at which CD4^+^ responses could be reproducibly obtained) or media only as a negative control, in 96-well ELISpot plates, and the levels of IFNγ produced by CD4^+^ T cells were determined in an ELISpot assay as previously described [8]. Cell culture supernatants were removed from the ELIspot assay at 72 h. All supernatants were frozen at ^−^80°C. Levels of IL-5, IL-9, IL-10, IL-13, IL-17, and tumor necrosis factor alpha (TNFα) were quantified following dilution 1:1 with AIM-V media. The bead assay, based on a capture sandwich immunoassay method, was adapted from the manufacturer’s protocol for a Bioplex assay (Bio-Rad). Briefly, a mixture of antibodies to the cytokines, coupled to internally dyed beads, were incubated with the samples and a standard curve generated by serial dilution of reconstituted standard. The plates were washed twice with commercial Luminex wash buffer, and biotinylated detection antibodies were added. Streptavidin-phycoerythrin was then added, and the beads were read using the Luminex 200 system (Luminex Corporation). The individual dyed bead populations as well as the fluorescent signal on the bead surface were detected. This allowed identification of each cytokine and reported the level of target protein in the well, extrapolated from the standard curve. IL-22 was quantified by ELISA following manufacturer’s directions (eBioscience), plates were read in a μQuant ELISA plate reader (BIO-Tek Instruments Inc.) using KC Junior software at a 450 nm wavelength with a reference wavelength of 630 nm. The cytokine concentration in the samples was extrapolated from the standard curve and expressed for all cytokines as Δ pg/ml concentration (pg/ml cytokine produced in response to LF domain IV - pg/ml cytokine produced in response to negative control). Analyses of the levels of each cytokine produced by the naturally infected, AVP-vaccinated, and healthy control cohorts, in response to the LF antigens, was compared using a two-way ANOVA with Bonferroni post hoc testing. All statistical analyses were determined by Kruskal Wallis with Dunns multiple comparison test performed using GraphPad Prism.

## Results

**Figure 1:**
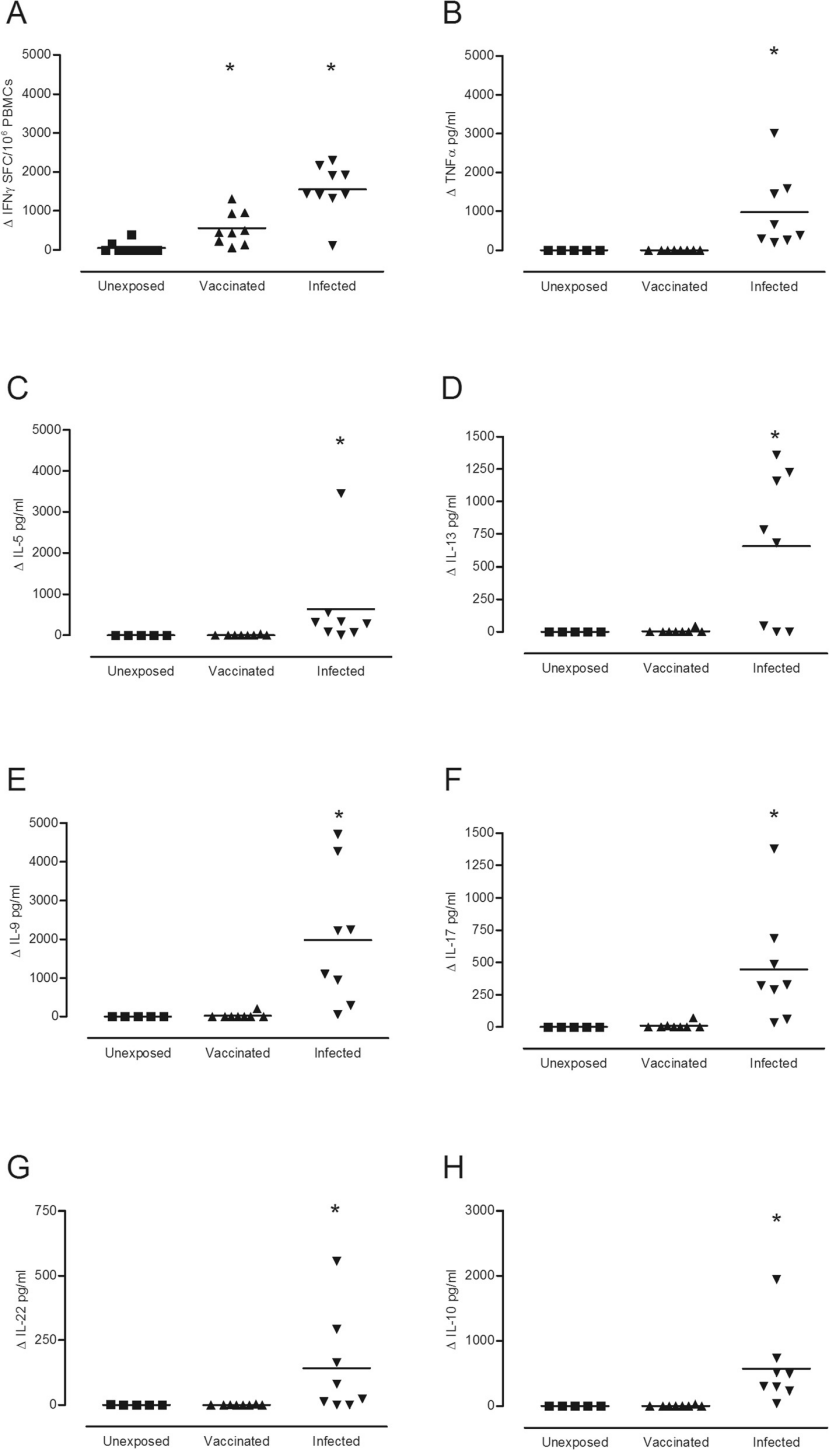
Differential cytokine responses to anthrax LF domain IV following cutaneous infection or AVP vaccination. Cells from individuals exposed to LF as a result of (▼) natural cutaneous infection (n = 8–9), or (▲) AVP vaccination (n = 8–10) and (■) unexposed healthy controls (n = 5-10) were stimulated with 25 μg/ml of LF domain IV *in vitro*, and the cytokine profile of the supernatants assessed by either ELIspot, Luminex or ELISA. ELIspot results **(A)** are expressed as the mean ΔSpot Forming Cells (SFC)/10^6^ PBMCs (stimulated – unstimulated background level), while the ELISA and Luminex values are given as the mean Δpg/ml detected for **(B)** TNFα, **(C)** IL-5, **(D)** IL-13, **(E)** IL-9, **(F)** IL-17, **(G)** IL-22 and **(H)** IL-10. * denotes a significantly greater cytokine production in comparison to the unexposed controls (p ≤ 0.05), as determined by Kruskal Wallis with Dunns multiple comparison test performed using GraphPad Prism version 5.01 for Windows, GraphPad Software, La Jolla California USA.

Compared to non-infected, unvaccinated individuals, naturally acquired cutaneous anthrax induced a diverse, CD4+ T cell cytokine response, encompassing significant, antigen-specific release of IFNγ (p < 0.001), TNFα (p < 0.001), IL-5 (p < 0.001), IL-9 (p < 0.001), IL-10 (p < 0.001), IL-13 (p = 0.045), IL-17 (p = 0.002) and IL-22 (p = 0.03) (Figure 1). Thus, cutaneous anthrax induces a broad T cell memory response characterized not only by the presence of Th1 cytokines IFNγ and TNFα, but also Th2 (IL-5 and IL-13), Th17 (IL-17/IL-22), Th22 (IL-22) and Th9 (IL-9) cytokines and a potentially regulatory IL-10 response. In contrast to the infection specific memory response to LF domain IV, initial exposure to the same antigen in the context of the AVP vaccine, led to a focused Th1 IFNγ response. Vaccinees show significantly more IFNγ (p = 0.002) than control subjects (Figure 1), but no other cytokines were detected.

## Discussion

Evidence from *in vivo* models and studies with cell lines has given a somewhat equivocal picture of the cytokine response to anthrax antigens. The inhibitory effects of both LF and EF upon expression of the activation markers CD25 and CD69 and the secretion of the pro-inflammatory cytokines IL-2, IL-5, TNFα, and IFNγ by human T cells has been described *in vitro* [16,15]. Elevated transcription of TNF-α, IL-1α, IL-1β, IL-4, IL-6, CCL5, CXCL2 and KC have been observed in both murine anthrax challenge models and *in vitro* macrophages and monocytic cell lines exposed to anthrax antigens [26-30]. Conversely, murine lymphocytes have shown impaired TCR mediated cell activation and selective suppression of the cytokines IL-2, IL-3, IL-4, IL-5, IL-6, IL-10, IL-17, TNFα, IFNγ and GM-CSF from CD4+ T cells following exposure to LF [14]. However, the cellular immunity we have identified within the naturally infected humans indicates that, although in vitro exposure to anthrax antigens has been implicated in immune deviation towards both the Th2 and Th17 pathways [31,18], the human immune response to pathological anthrax exposure encompasses a cytokine profile associated with a broad range of Th subsets with little or no evidence of helper T cell polarization. Indeed, following anthrax infection, *in vitro* recall responses to the LF domain IV protein were characterised by a more diverse cytokine profile than immunization with the AVP vaccine was capable of provoking. The response to this immunogenic domain of LF was dominated by IFNγ release in the vaccinees, whilst the individuals exposed to LF following cutaneous anthrax infection showed significantly elevated levels of the pro-inflammatory cytokines in their *in vitro* recall response associated with Th1, Th2, Th9 and Th17 subsets, compared to vaccinees and naïve controls. Previous work has suggested that AVP vaccination has the capacity to lead to a suppressed Th1 and Th2 response to LF and PA, relative to the response mounted by naturally infected individuals [8]. This is the first work to examine in detail the effect of either encountering the antigen in the context of natural infection or vaccination upon the cytokine profile provoked by re-exposure to LF domain IV. Although this is the first study, to our knowledge, to implicate IL-22 in the host immune response against anthrax, recent analysis of the role of IL-22 in Th17 mediated host immunity to bacteria at barrier surfaces [32], demonstrates the importance of this cytokine in facilitating antimicrobial gene expression. In addition to the Th17 response, the known role of IL-22 and IL-17 in promoting Th1 immunity to bacterial pathogens [33] may play a crucial role in preventing the survival of *B. anthracis* within the host. Conversely, survival of *B. anthracis* in an unprotected host is dependent upon a rapid suppression of Th1 cytokines [3]. Whilst we previously reported that the patient who developed toxemic shock during *B. anthracis* infection showed the highest level of IFNγ responses to both PA and LF [8], examining the elevated cytokine profiles in these patients did not reveal a discernable trend related to either the period of time post infection, the duration of infection or the clinical severity.

The marked difference noted between the infected individuals and vaccinees echoes the divergence in the epitope repertoire recognised by each cohort. Whilst it might be expected that some epitopes present in the context of vaccination would be lost upon infection [34], the immune response detected after AVP immunization differed substantially from that following infection [9,8]. It is unclear whether this represents the differential antigen processing of pathogen associated proteins experienced in vaccination in contrast to infection, or if it represents an artefact of the repeated AVP vaccinations which may have served to skew the cytokine environment present during the induction of the immune response, impacting upon the T cell epitope repertoire [35]. Conversely, the difference both in the epitopes recognised and the nature of cytokine responses between the vaccinated and infected groups may relate to the route of antigen exposure, as natural infection was localised to the skin, in contrast to intra-muscular vaccination. In the skin, bacterial antigens are processed and presented by different subsets of dendritic cells [36], increasing the potential for induction of a variety of Th responses. Alternatively, the diversity of this response may represent the complex interaction of the immune system with anthrax toxins and a live, dividing bacterium where exposure duration is perhaps more prolonged. The divergence we describe in the immune response post-infection, compared to vaccination, is not unprecedented; *Mycobacterium tuberculosis* infection results in high levels of mycobacteria-specific IL-17 [37] and IL-9 [38] produced by T cells, whereas in recent clinical trials of the MVA85A vaccine, only extremely high doses induced a significant increase in IL-17 production, despite prior BCG vaccination [39].

The human immune response to natural bacterial infection is often more complex than has been shown in murine infection models and the existing vaccines are less well-defined than recombinant sub-unit vaccines now in clinical trial. It will be interesting to examine the T-cell responses induced in human vaccines by these defined recombinant anthrax vaccines to determine if a cytokine profile associated with protection from lethal anthrax infection is induced.

## Abbreviations

APC: Antigen presenting cell
AVP: Anthrax vaccine precipitated
DC: Dendritic cell
EF: Edema factor
ELISA: Enzyme linked immunosorbent assay
ELISpot: Enzyme linked immunospot
IFN: Interferon
IL: Interleukin
LF: Lethal factor
MAPK: Mitogen-activated protein kinase
PA: Protective antigen
PBMC: Peripheral blood mononuclear cells
Th: T helper
TNFα: Tumor necrosis factor alpha
